# AI literacy as a potential mediator between attitude and self-efficacy among PICU nurses: a cross-sectional study

**DOI:** 10.3389/fpubh.2026.1812848

**Published:** 2026-05-15

**Authors:** Yu Liu, Xiufang Zhao, Qin Zeng

**Affiliations:** 1 Department of Pediatric Intensive Care Unit Nursing, West China Second University Hospital, Sichuan University/West China School of Nursing, Sichuan University, Chengdu, China; 2 Key Laboratory of Birth Defects and Related Diseases of Women and Children (Sichuan University), Ministry of Education, Chengdu, China; 3 Department of Nursing, West China Second University Hospital, Sichuan University, Chengdu, China; 4 Department of Pediatrics Gastroenterology Nursing, West China Second University Hospital, Sichuan University, Chengdu, China

**Keywords:** AI literacy, artificial intelligence, mediation analysis, nursing management, pediatric intensive care unit (PICU), self-efficacy

## Abstract

**Background:**

Artificial intelligence (AI) is reshaping the healthcare landscape, particularly in high-stakes settings like pediatric intensive care units (PICU). While AI holds potential to enhance clinical care, its integration into nursing practice may depend on nurses’ attitudes, literacy, and self-efficacy regarding AI. However, the relationships among these constructs—and whether AI literacy plays an indirect role in the attitude–self-efficacy association—remain underexamined in PICU nurses.

**Aim:**

This cross-sectional study examined whether AI literacy is statistically consistent with a mediating role in the relationship between PICU nurses’ AI attitude and AI self-efficacy.

**Design:**

A multicenter cross-sectional study conducted in Sichuan Province, China.

**Methods:**

A convenience sample of 221 registered nurses from 10 PICUs in one Chinese province completed self-report measures of the General Attitudes toward Artificial Intelligence Scale (GAAIS), the Artificial Intelligence Literacy Scale (AILS), and the Artificial Intelligence Self-Efficacy Scale (AISES). Data analyses included Pearson correlation and mediation analysis (PROCESS Model 4) with 5,000 bootstrap samples. Given the cross-sectional design, all analyses are associational, not causal.

**Results:**

PICU nurses reported generally positive AI attitude, AI literacy, and AI self-efficacy on the respective scales. Positive correlations were observed among all three variables (all *p* < 0.01), with the strongest association observed between AI literacy and self-efficacy (*r* = 0.743). The indirect effect of AI attitude on AI self-efficacy via AI literacy was statistically consistent with mediation [indirect effect = 0.815, 95% CI (0.582, 1.082)], accounting for 76% of the total effect. Regarding subgroup differences, nurses with advanced degrees and those employed in teaching hospitals showed higher mean scores in AI literacy and self-efficacy (*p* < 0.05).

**Conclusion:**

In this cross-sectional sample of PICU nurses, the findings were consistent with an indirect role of AI literacy in the association between AI attitude and AI self-efficacy. However, because the data are cross-sectional, causality cannot be inferred. These results are hypothesis-generating and support future longitudinal and intervention studies to test whether enhancing AI literacy can improve AI self-efficacy over time. Pending such confirmation, nursing administrators may consider integrating AI literacy into continuing education as one potential strategy to support nurses’ AI self-efficacy.

## 1 Introduction

Artificial intelligence (AI), characterized by its capabilities in machine learning, reasoning, perception, and decision-making, has been applied across numerous sectors, including healthcare (1–3). Within medical systems, AI technologies may augment clinical practice by supporting diagnostic precision, informing treatment regimens, and assisting with complex clinical workflows (1–3). Nurses constitute the largest professional group within healthcare and are therefore a critical component for the integration of AI (4). A growing body of evidence suggests that AI applications in nursing are associated with improvements in the accuracy, efficiency, and safety of clinical practice (5, 6). This is especially evident in high-acuity environments like the Pediatric Intensive Care Unit (PICU). Studies show that AI can leverage predictive models to identify health risks in critically ill children, reduce mortality, and offer real-time decision support (7–9). Moreover, AI-powered clinical decision support systems have been associated with improvements in both the quality of care and workflow efficiency within the PICU (10).

Nevertheless, the deployment of AI also raises concerns regarding algorithmic bias, data privacy, information security, and excessive technological reliance (6). In the context of nursing, over-dependence on AI could potentially erode the foundational critical thinking and clinical judgment of nurses. This reality underscores that the integration of AI hinges not merely on technological sophistication but also on nurses’ attitudes, comprehension, and their ability to apply AI in practice—a concept related to AI self-efficacy. While nurses generally report positive attitudes toward AI, this coexists with gaps in their understanding of its core principles, applications, and ethical guidelines (11). Recent systematic reviews have synthesized evidence indicating that nurses and nursing students exhibit “guarded optimism” toward AI, with AI literacy and self-efficacy being key predictors of adoption, while anxiety reduces readiness (12). A meta-analysis reported that approximately 73% of nurses demonstrate AI awareness and 71% hold positive attitudes, yet major barriers to AI adoption include the absence of formal AI curricula, limited faculty expertise, inadequate infrastructure, and concerns over data privacy and algorithmic bias (13, 14). This disconnect between attitude and comprehension highlights a gap in the literature: a lack of understanding regarding whether and how positive attitudes are associated with AI self-efficacy. To this end, this study is grounded in Bandura’s Social Cognitive Theory (SCT) (15). SCT posits a dynamic interplay between personal cognitive factors, behavior, and environmental influences, assigning central importance to self-efficacy in shaping learning and behavioral outcomes. This theoretical framework aligns with a nursing consensus that calls for developing “minimum AI in nursing competencies” and integrating AI literacy into nursing curricula (16). To explore this framework, we examined the statistical consistency of a mediation model in which AI literacy is hypothesized to play an indirect role linking AI attitude to AI self-efficacy. We hypothesize that a positive AI attitude may encourage nurses to acquire AI-related knowledge and skills, thereby being associated with higher AI literacy. This elevated literacy, in turn, may provide a basis for greater confidence in applying AI technologies clinically—that is, it may be associated with stronger AI self-efficacy.

Understanding the relationship among these constructs is relevant within China’s regional healthcare contexts. The rapid integration of AI into China’s health system may be reshaping nursing (17), but its benefits are not uniformly distributed. Regional inequalities in infrastructure, resource allocation, and professional development opportunities pose a barrier to nurses’ AI adoption, potentially compounding the digital divide. A case in point is Yunnan Province, where hospitals demonstrably lag behind Beijing in both AI application levels and the availability of nurse training resources (18). Consequently, examining the associations between PICU nurses’ AI attitudes, literacy, and self-efficacy may help identify implementation barriers in resource-limited contexts. These insights may inform nursing leaders in designing strategies that aim to enhance AI readiness and facilitate the integration of AI into clinical care.

Therefore, this study examined the hypothesis that AI literacy is statistically consistent with a mediating role in the relationship between AI attitude and self-efficacy among PICU nurses in Sichuan Province, China. We tested the following specific hypotheses:

*H1*: AI attitude is positively correlated with AI self-efficacy.

*H2*: AI literacy shows a statistically significant partial indirect effect between AI attitude and AI self-efficacy.

## 2 Participants and methods

### 2.1 Study design

A cross-sectional survey was conducted between May 1 and July 1, 2025, to investigate the relationships among AI attitude, AI literacy, and AI self-efficacy among registered nurses working in pediatric intensive care units (PICU) of public hospitals in Sichuan Province, China. A convenience sampling method was employed to recruit participants. *A priori* sample size estimation was performed using G*Power software. With an alpha of 0.05, a power of 0.80, and an effect size of 0.05, a minimum of 193 participants was required. Accounting for an anticipated 10% dropout rate, the target sample size was set at 215 (19).

### 2.2 Study participants

Registered nurses were recruited from the pediatric intensive care units (PICU) of public hospitals in Sichuan Province. The inclusion criteria required participants to be: (1) registered nurses working in a PICU; (2) possess a minimum of 1 year of clinical experience; and (3) provide voluntary informed consent. Participants were excluded if they: (1) completed the survey in less than 120 s; (2) had prior involvement in a similar survey study; or (3) were on extended leave (exceeding 6 months).

### 2.3 Study instruments

#### 2.3.1 Demographic and work-related questionnaire

A self-administered questionnaire was used to collect data on demographic and work-related characteristics. The collected variables included gender, age, education level, professional title, years of clinical experience, primary job duties, preceptor role, prior AI training, hospital type and level, and teaching hospital status. To specifically gauge nurses’ acceptance of AI, two additional items were included: “Are you willing to use AI to assist your learning and work?” and “Are you willing to receive AI-related knowledge training?”. Responses were captured on an 11-point Likert scale ranging from 0 (“completely unwilling”) to 10 (“completely willing”).

#### 2.3.2 General Attitudes Toward Artificial Intelligence Scale (GAAIS)

The General Attitudes Toward Artificial Intelligence Scale (GAAIS), developed by Schepman and Rodway (20), was utilized to assess PICU nurses’ overall attitudes toward AI. The scale has been validated for effectively measuring healthcare professionals’ positive and negative attitudes toward AI (18) and has been widely used and validated among nursing staff and student populations (18, 19, 21, 22). The scale comprises 20 items, divided into two subscales: Positive Attitude (12 items, Items 1–12) and Negative Attitude (8 items, Items 13–20). Responses are captured on a 5-point Likert scale (1 = “Strongly Disagree” to 5 = “Strongly Agree”). Items within the Negative Attitude subscale are reverse scored. The total score ranges from 20 to 100, with higher scores indicating a more positive attitude toward AI. In the original validation study, the Cronbach’s alpha coefficients were 0.880 for the Positive Attitude subscale and 0.830 for the Negative Attitude subscale. In the present study, the internal consistency reliability was high, with Cronbach’s alpha values of 0.950 for the Positive Attitude subscale, 0.956 for the Negative Attitude subscale, and 0.902 for the total scale, indicating the instrument’s high reliability within our sample. In our sample, the KMO values for the positive subscale, negative subscale, and total scale were 0.937, 0.916, and 0.918, respectively (Bartlett’s test *p* < 0.001).

#### 2.3.3 Artificial Intelligence Literacy Scale (AILS)

The Artificial Intelligence Literacy Scale (AILS), developed by Wang et al. (23) was used to assess PICU nurses’ AI literacy. This scale has been validated in both student and nurse populations, demonstrating sound psychometric properties (24–26). It is founded on a four-dimensional model, comprising Awareness (3 items), Usage (3 items), Evaluation (3 items), and Ethics (3 items), for a total of 12 items. Responses were recorded on a 7-point Likert scale (1 = Strongly Disagree to 7 = Strongly Agree), with specific items (Awareness item 2, Usage item 2, Ethics item 2) being reverse-scored. The total score ranges from 12 to 84, with higher scores indicating a higher level of AI literacy. In the present study, the AILS showed high internal consistency, with a Cronbach’s alpha of 0.836, comparable to the original validation coefficient of 0.830. In our sample, the KMO value for the total AILS was 0.895 (Bartlett’s test *p* < 0.001).

#### 2.3.4 Artificial Intelligence Self-Efficacy Scale (AISES)

This study employed the Artificial Intelligence Self-Efficacy Scale (AISES) developed by Wang and Chuang (27) to assess PICU nurses’ AI self-efficacy levels. This scale measures individuals’ self-efficacy when using AI technologies or products and has been validated and applied among students, educators, and nursing populations (18, 28, 29). The AISES comprises 22 items organized into four dimensions: Assistance (7 items), Anthropomorphic Interaction (5 items), Comfort with AI (6 items), and Technological Skills (4 items). The scale employs a 7-point Likert scale (1 = “Strongly Disagree,” 7 = “Strongly Agree”), with total scores ranging from 22 to 154. Higher scores indicate greater AI self-efficacy among participants. In the original study, the Cronbach’s *α* coefficients for the AISES total scale and its dimensions were 0.958, 0.942, 0.970, 0.963, and 0.869, respectively, demonstrating excellent internal consistency and reliability. In this study, the Cronbach’s α coefficient for the AISES scale was 0.984, indicating excellent internal consistency and measurement stability of the instrument within this sample. In our sample, the KMO value for the total AISES was 0.952 (Bartlett’s test *p* < 0.001).

### 2.4 Data collection and quality control

Data were collected electronically via Wenjuanxing,^1^ a prevalent online survey platform in China known for its security and anonymity. Participants accessed the survey via a QR code and provided electronic informed consent before proceeding. The questionnaire featured standardized instructions, mandatory items, and was paginated to prevent omissions. To ensure data integrity, each IP address was limited to a single submission, and all responses were anonymized upon automatic export.

The research team monitored response quality in real-time to identify duplicates or anomalies. Following data export, two researchers independently cross-checked the dataset, excluding responses with implausible completion times, irrational answers, or logical inconsistencies. The resultant high-quality dataset was thus prepared for analysis.

### 2.5 Statistical analysis

Data management was performed in Excel, and statistical analyses were conducted using SPSS 27.0. Descriptive statistics are presented as frequencies (percentages) for categorical variables and mean ± standard deviation for continuous variables.

Normality of continuous variables was examined using skewness and kurtosis values. For AI attitude (skewness = 0.964, kurtosis = 0.520), AI literacy (skewness = 0.864, kurtosis = 0.300), and AI self-efficacy (skewness = 0.438, kurtosis = −0.969), all absolute values were within the acceptable range (skewness < 2, kurtosis < 7), indicating approximate normality. Homogeneity of variances was assessed using Levene’s test. When the assumption was violated, Welch’s t-test (for two-group comparisons) or Welch’s *F*-test (for multi-group comparisons) was reported.

Multicollinearity among the predictor variables (AI attitude, AI literacy, education level, professional title, and teaching hospital status) was assessed using variance inflation factor (VIF). VIF values ranged from 1.061 to 1.574, indicating no concern for multicollinearity.

Group comparisons for continuous outcomes involved independent t-tests (for binary predictors) or one-way ANOVA (for categorical predictors), applied when normality and homogeneity of variance assumptions were satisfied. Associations between continuous variables were examined using Pearson correlation.

To assess the potential threat of common method bias, we conducted Harman’s single-factor test. All items from the GAAIS, AILS, and AISES were entered into an unrotated exploratory factor analysis. The results showed that the first factor accounted for 49.23% of the total variance. According to the rationale of Harman’s single-factor test (30), no single general factor emerged that accounts for the majority of the covariance among the measures, suggesting that common method bias is unlikely to be a severe concern in this study.

Given the number of comparisons performed (33 comparisons in Table 1), a conservative alpha level of *p* < 0.01 was used to interpret statistical significance for subgroup analyses to reduce the risk of Type I error. Nominally significant findings at *p* < 0.05 were considered exploratory and interpreted with caution.

**Table 1:** Univariate analyses of study variables by sociodemographic characteristics.

Variable	AI attitude (*M* ± SD)	AI literacy (*M* ± SD)	AI self-efficacy (*M* ± SD)
Gender			
Male	71.30 ± 9.62	55.90 ± 6.61	114.50 ± 27.40
Female	71.00 ± 11.06	58.00 ± 9.48	112.90 ± 22.79
*t*	0.083	−0.690	0.216
*p*	0.934	0.491	0.830
Age group (years)			
≤30	71.00 ± 12.03	58.33 ± 9.69	115.17 ± 24.66
31–40	71.65 ± 10.85	58.17 ± 9.44	113.62 ± 22.22
41–50	69.56 ± 9.39	56.83 ± 8.87	107.35 ± 20.99
51–60	76.00 ± 7.17	55.75 ± 8.50	119.00 ± 15.88
*F*	0.642	0.360	1.345
*p*	0.589	0.782	0.261
Educational level			
Associate degree or below	69.65 ± 10.12	56.60 ± 8.86	109.65 ± 23.92
Bachelor’s degree	71.08 ± 11.34	57.72 ± 9.34	112.74 ± 22.60
Master’s degree or above	75.38 ± 8.69	64.92 ± 9.14	128.00 ± 18.93
*F*	1.411	4.267	3.370
*p*	0.246	0.015	0.036
Professional title			
Junior	71.06 ± 11.55	58.46 ± 9.66	112.29 ± 24.06
Intermediate	70.23 ± 10.46	56.23 ± 8.91	111.21 ± 21.33
Senior	73.74 ± 8.70	60.53 ± 8.38	124.37 ± 18.71
*F*/Welch’s *F*	0.769	2.154	3.743
*p*	0.456	0.118	0.030
Years of work experience			
< 5	68.94 ± 8.55	57.22 ± 8.54	110.96 ± 23.62
6–10	73.96 ± 13.17	59.48 ± 10.97	116.21 ± 25.67
11–15	69.20 ± 10.14	56.98 ± 9.84	110.11 ± 20.42
≥16	71.90 ± 11.04	57.97 ± 8.10	114.48 ± 22.22
*F*/Welch’s *F*	2.376	0.762	0.841
*p*	0.073	0.516	0.474
Job content			
Clinical nursing	71.04 ± 11.10	58.07 ± 9.48	112.24 ± 23.20
Administration/teaching/research/other	70.82 ± 10.05	56.36 ± 8.26	119.55 ± 19.86
*t*	0.090	0.810	−1.420
*p*	0.929	0.419	0.157
Clinical educator status			
Yes	71.84 ± 11.94	58.22 ± 9.71	114.86 ± 22.48
No	69.95 ± 9.54	57.49 ± 8.93	110.51 ± 23.44
*t*	1.272	0.571	1.398
*p*	0.205	0.569	0.163
AI-related training			
Received formal training	69.46 ± 12.79	59.67 ± 11.84	117.38 ± 22.71
Informal training/self-study	71.03 ± 10.53	58.06 ± 9.44	112.66 ± 22.94
No training at all	71.38 ± 11.02	57.44 ± 8.66	112.22 ± 23.13
*F*	0.294	0.628	0.503
*p*	0.745	0.534	0.605
Hospital type			
Specialized hospital	71.98 ± 10.98	57.97 ± 9.56	114.36 ± 22.92
General hospital	69.87 ± 10.92	57.82 ± 9.18	111.32 ± 23.00
*t*	1.428	1.114	0.981
*p*	0.155	0.909	0.328
Hospital grade			
Tertiary A	71.77 ± 11.18	58.20 ± 9.79	114.43 ± 23.53
Tertiary B	68.79 ± 7.80	56.56 ± 7.37	111.44 ± 20.89
Secondary and below	69.27 ± 12.90	57.81 ± 9.14	105.88 ± 21.14
*F*	1.411	0.430	1.652
*p*	0.246	0.651	0.194
Teaching hospital			
Yes	72.06 ± 11.06	58.37 ± 9.57	115.20 ± 23.72
No	67.63 ± 10.07	56.37 ± 8.58	105.71 ± 18.68
*t*	2.574	1.354	2.995
*p*	0.011	0.177	0.003

Data are presented as mean ± standard deviation. *t*-values represent independent-samples *t*-tests; *F*-values represent one-way ANOVA. Welch’s *F* is reported for professional title (AI self-efficacy) and work experience (AI attitude and self-efficacy) due to violation of homogeneity of variance. Given 33 comparisons, a conservative threshold of *p* < 0.01 was used to interpret significance; *p* < 0.05 findings are exploratory.

The hypothesized mediation model, in which AI literacy was hypothesized to play an indirect role in the relationship between AI attitude and AI self-efficacy, was tested using the PROCESS macro for SPSS (Model 4) with 5,000 bootstrap samples. Based on Bandura’s Social Cognitive Theory, which posits that personal factors (e.g., education, professional experience) and environmental factors (e.g., institutional resources) influence self-efficacy, the model was adjusted for covariates including education level, professional title, and teaching hospital status. These covariates were selected because they showed associations with AI self-efficacy in bivariate analyses (*p* < 0.05; Table 1). The indirect effect was considered statistically significant if its 95% bias-corrected confidence interval did not include zero. The significance level for all tests was set at *α* = 0.05 (two-tailed).

## 3 Results

### 3.1 Demographic characteristics and AI attitude, literacy, and self-efficacy scores

The demographic and work-related characteristics of the 221 PICU nurses from 10 hospitals are summarized in Table 2. The cohort was predominantly female (95.5%), under 40 years old (77.9%), and held at least a bachelor’s degree (78.3%). While 90.0% were primarily clinical staff, a majority (56.6%) also held preceptor roles. Formal AI training was uncommon, with only 10.9% having received it. High willingness was reported for both using AI (8.76 ± 1.85) and receiving AI training (8.65 ± 1.88).

**Table 2:** Sociodemographic characteristics of participants (*N* = 221).

Characteristics	Frequency/Mean	Percentage/SD
Gender		
Male	10	4.5%
Female	211	95.5%
Age group (years)		
≤30	87	39.4%
31–40	85	38.5%
41–50	45	20.4%
51–60	4	1.8%
Educational level		
Associate degree or below	48	21.7%
Bachelor’s degree	160	72.4%
Master’s degree or above	13	5.9%
Professional title		
Junior	129	58.4%
Intermediate	73	33.0%
Senior	19	8.6%
Years of work experience		
≤5	51	23.1%
6–10	52	23.5%
11–15	56	25.3%
≥16	62	28.1%
Job content		
Clinical nursing	199	90.0%
Administration/teaching/research/other	22	10.0%
Clinical educator status		
Yes	125	56.6%
No	96	43.4%
AI-related training		
Received formal training	24	10.9%
Informal training/self-study	96	43.4%
No training at all	101	45.7%
Hospital type		
Specialized hospital	120	54.3%
General hospital	101	45.7%
Hospital grade		
Tertiary A	161	72.9%
Tertiary B	34	15.4%
Secondary and below	26	11.8%
Teaching hospital		
Yes	169	76.5%
No	52	23.5%
Willingness to Use AI to assist in learning and work	8.76^a^	1.85^b^
Willingness to receive training on AI-related knowledge	8.65^a^	1.88^b^

a Mean.

b Standard Deviation.

Table 3 displays the item-level scores for AI attitude, literacy, and self-efficacy. PICU nurses reported mean scores of AI attitude (GAAIS: 71.02 ± 10.98), AI literacy (AILS: 57.90 ± 9.37), and AI self-efficacy (AISES: 112.97 ± 22.95). Notably, the highest-scoring items were “I am eager to use AI systems in daily life” (P_5: 3.94 ± 0.75) and “AI has many beneficial applications” (P_9: 3.91 ± 0.74), reflecting strong recognition of AI’s value. Conversely, concerns about reliability were evident in items like “I believe AI systems make many mistakes” (N_2: 3.09 ± 0.96). Furthermore, the greatest heterogeneity among the scales was observed for self-efficacy, as indicated by its large standard deviation.

**Table 3:** Descriptive statistics of item scores for AI attitude, AI literacy, and AI self-efficacy among PICU nurses (*N* = 221).

Item	Description	Mean	SD
The General Attitudes towards Artificial Intelligence Scale (GAAIS)			
P_1	For routine transactions, I would rather interact with an artificially intelligent system than with a human	3.20	0.99
P_2	Artificial Intelligence can provide new economic opportunities for this country	3.93	0.83
P_3	Artificially intelligent systems can help people feel happier	3.76	0.78
P_4	I am impressed by what Artificial Intelligence can do	3.69	0.82
P_5	I am interested in using artificially intelligent systems in my daily life	3.94	0.75
P_6	Artificial Intelligence can have positive impacts on people’s wellbeing	3.87	0.82
P_7	Artificial Intelligence is exciting	3.73	0.80
P_8	An artificially intelligent agent would be better than an employee in many routine jobs	3.49	0.90
P_9	There are many beneficial applications of Artificial Intelligence	3.91	0.74
P_10	Artificially intelligent systems can perform better than humans	3.52	0.89
P_11	Much of society will benefit from a future full of Artificial Intelligence	3.82	0.79
P_12	I would like to use Artificial Intelligence in my own job	3.86	0.80
Negative Attitudes			
N_1	Organizations use Artificial Intelligence unethically	3.18	1.18
N_2	I think artificially intelligent systems make many errors	3.09	0.96
N_3	I find Artificial Intelligence sinister	3.43	1.11
N_4	Artificial Intelligence might take control of people	3.28	1.11
N_5	I think Artificial Intelligence is dangerous	3.27	1.02
N_6	I shiver with discomfort when I think about future uses of Artificial Intelligence	3.45	1.05
N_7	People like me will suffer if Artificial Intelligence is used more and more	3.41	1.04
N_8	Artificial Intelligence is used to spy on people	3.19	1.03
Artificial Intelligence Literacy Scale (AILS)			
AW_1	I can distinguish between smart devices and non-smart devices	5.12	1.23
AW_2	I do not know how AI technology can help me	3.82	1.53
AW_3	I can identify the AI technology employed in the applications and products I use	4.97	1.21
US_1	I can skillfully use AI applications or products to help me with my daily work	4.82	1.30
US_2	It is usually hard for me to learn to use a new AI application or product^R^	4.18	1.54
US_3	I can use AI applications or products to improve my work efficiency	5.28	1.41
EV_1	I can evaluate the capabilities and limitations of an AI application or product after using it for a while	5.03	1.20
EV_2	I can choose a proper solution from various solutions provided by a smart agent	5.07	1.20
EV_3	I can choose the most appropriate AI application or product from a variety for a particular task	5.11	1.74
ET_1	I always comply with ethical principles when using AI applications or products	5.44	1.16
ET_2	I am never alert to privacy and information security issues when using AI applications or products ^R^	3.98	1.63
ET_3	I am always alert to the abuse of AI technology	5.09	1.23
Artificial Intelligence Self-Efficacy Scale (AISES)			
AS_1	Some AI technologies/products make learning easier	5.49	1.08
AS_2	I find that AI technologies/products are helpful for learning	5.50	1.06
AS_3	AI technologies/products are good aids to learning	5.54	1.04
AS_4	Using AI technologies/products makes learning more interesting	5.49	1.05
AS_5	I’m confident in my ability to learn simple programming of AI technologies/products if I were provided the necessary training	5.25	1.18
AS_6	AI technologies/products help me to save a lot of time	5.45	1.03
AS_7	I find it easy to get AI technologies/products to do what I want it to do	5.35	1.09
AI_1	I think the interactive process of AI technologies/products is very vivid, just like chatting with a real person	5.25	1.17
AI_2	I think the way that AI technologies/products express content when interacting is unique, just like a real person	5.10	1.21
AI_3	I think there is no difference between the dialogue method of AI technologies/products compared with the dialogue with real people	4.81	1.45
AI_4	I think the tone of AI technologies/products when interacting is the same as that of real people	4.76	1.46
AI_5	I feel that the way of expression of AI technologies/products in the interactive text is the same as that of real people	4.81	1.42
CF_1	When interacting with AI technologies/products, I feel very calm	5.14	1.16
CF_2	When interacting with AI technologies/products, I find it easy	5.14	1.13
CF_3	When interacting with AI technologies/products, I feel comfortable in my heart	5.08	1.16
CF_4	When interacting with AI technologies/products, I feel very peaceful	5.00	1.19
CF_5	When interacting with AI technologies/products, I feel very relaxed	5.14	1.14
CF_6	I can happily interact with AI technologies/products smoothly	5.14	1.14
TS_1	When using AI technologies/products, I am not worried that I might press the wrong button and cause risks	4.89	1.31
TS_2	When using AI technologies/products I am not worried that I might press the wrong button and damage it	4.83	1.35
TS_3	When using an AI technology/product, there is nothing that I do not know why	4.88	1.34
TS_4	AI technologies/products jargon does not baffle me	4.92	1.25

R indicates that the item is reverse scored. P, Positive Attitudes; N, Negative Attitudes; AW, Awareness; US, Usage; EV, Evaluation; ET, Ethics; AS, Assistance; AI, Anthropomorphic Interaction; CF, Comfort with AI; TS, Technological Skills.

### 3.2 Comparison of AI attitude, literacy, and self-efficacy by participant characteristics

Table 1 summarizes the comparisons across demographic and work-related characteristics. No significant differences were found in relation to gender, age, job role, preceptor status, AI training, hospital type, or grade (all *p* > 0.05). Exploratory analyses revealed that nurses with a master’s degree or higher demonstrated higher AI literacy (*F* = 4.267, *p* = 0.015) and self-efficacy (*F* = 3.370, *p* = 0.036) compared to other groups. Exploratory analyses also showed that professional title was associated with AI self-efficacy after applying Welch’s *F*-test (Welch’s *F* = 3.743, *p* = 0.030). Additionally, exploratory analyses indicated that nurses in teaching hospitals reported higher AI attitude scores (*t* = 2.574, *p* = 0.011). However, a significant difference was observed for AI self-efficacy by teaching hospital status (*t* = 2.995, *p* = 0.003), which met the conservative threshold of *p* < 0.01.

### 3.3 Correlations among AI attitude, literacy, self-efficacy, and willingness

Pearson correlation analysis was conducted to examine the interrelationships among the study variables (Table 4). AI attitude was positively associated with both AI literacy (*r* = 0.589, *p* < 0.001) and AI self-efficacy (*r* = 0.533, *p* < 0.001). AI literacy demonstrated robust positive correlations with self-efficacy (*r* = 0.743, *p* < 0.001), willingness to use AI (*r* = 0.381, *p* < 0.001), and willingness to receive AI training (*r* = 0.348, *p* < 0.001). Similarly, AI self-efficacy was significantly correlated with both willingness to use AI (*r* = 0.445, *p* < 0.001) and willingness to receive AI training (*r* = 0.375, *p* < 0.001). Notably, the strongest correlation observed was between willingness to use AI and willingness to receive AI training (*r* = 0.852, *p* < 0.001). All correlations were statistically significant.

**Table 4:** Correlations among AI attitude, AI literacy, AI self-efficacy, and willingness to use and receive training (*N* = 221).

Variable	Mean	SD	1	2	3	4	5
1. AI Attitude	71.02	10.98	1				
2. AI Literacy	57.90	9.37	0.589**	1			
3. AI Self-Efficacy	112.97	22.95	0.533**	0.743**	1		
4. Willingness to Use AI	8.76	1.85	0.439**	0.381**	0.445**	1	
5. Willingness to Receive AI Training	8.65	1.88	0.458**	0.348**	0.375**	0.852**	1

**p < 0.001 (two-tailed).

### 3.4 Mediation analysis of AI literacy in the attitude-self-efficacy relationship

**Figure 1:**
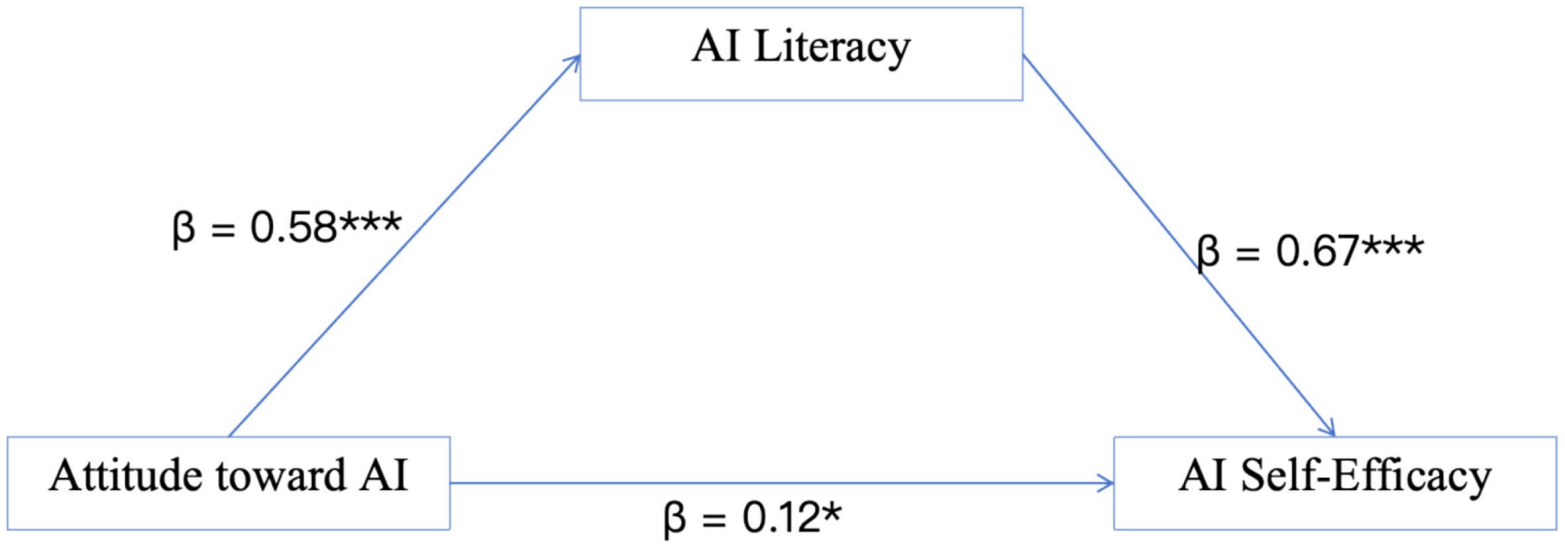
Mediation analysis of AI attitude, AI literacy, and AI self-efficacy among PICU nurses. Arrows represent statistical associations, not causal pathways. The model was adjusted for education level, professional title, and teaching hospital status. ***p < 0.001, *p < 0.05.

A mediation analysis, adjusted for covariates including education level, professional title, and teaching hospital status, was conducted to examine the hypothesized indirect role of AI literacy between AI attitude and AI self-efficacy (Figure 1). The total effect of attitude on self-efficacy was significant (*β* = 0.51, *p* < 0.001). When AI literacy was included as a mediator, the direct effect remained significant but was notably smaller (*β* = 0.12, *p* = 0.028), which is consistent with a partial indirect effect. The indirect effect via AI literacy was significant [*β* = 0.39, 95% CI (0.28, 0.51)]. As detailed in Table 5, this indirect effect accounted for approximately 76% of the total effect, suggesting that AI literacy plays a substantial indirect role in the relationship between AI attitude and AI self-efficacy among PICU nurses.

**Table 5:** Mediation effect analysis of AI attitude on AI self-efficacy through AI literacy.

Effect type	*β*	SE	LLCI	ULCI	Effect ratio
Total effect	0.5118	0.0589	0.3955	0.6295	–
Direct effect	0.1220	0.0551	0.0140	0.2302	23.8%
Indirect effect	0.3898	0.0591	0.2800	0.5112	76.2%

## 4 Discussion

### 4.1 Current status of AI attitude, literacy, and self-efficacy

This investigation revealed that PICU nurses in Sichuan Province reported mean scores of AI attitude (71.02 ± 10.98), AI literacy (57.90 ± 9.37), and AI self-efficacy (112.97 ± 22.95). The overall AI attitude score is comparable to findings reported by Tuncer and Tuncer (31). However, scores on selected negative attitude items (e.g., item N_2: 3.09 ± 0.96) suggest some concerns regarding AI reliability. This tension between recognition of AI’s utility and ethical or safety concerns has been noted in the literature on healthcare technology adoption (6). These findings are hypothesis-generating and suggest that future research could explore whether interventions addressing both AI-related technical skills and ethical concerns may be beneficial for AI integration in nursing.

### 4.2 Influence of demographic characteristics on AI attitude, literacy, and self-efficacy

#### 4.2.1 Impact of nurses’ AI cognition and training

This study reveals a notable gap in AI preparedness among PICU nurses: while attitudes toward AI training are generally positive, access to formal training is limited, with only 10.9% having received specialized AI instruction. This finding is consistent with previous reports of low training rates among nurses (2, 29), highlighting a persistent gap between willingness and opportunity. This scarcity of formal training may contribute to the observed moderate levels of AI literacy, suggesting room for improvement in AI knowledge and clinical application skills (2, 32).

Future initiatives may need to consider establishing a systematic knowledge base through integrating AI literacy into nursing curricula and fostering operational confidence through increased hands-on experience with AI tools. For resource-limited regions such as Sichuan, ensuring equitable access to AI training remains an important consideration. However, the specific effectiveness of online platforms, inter-institutional partnerships, or virtual simulations requires further investigation in longitudinal or experimental studies.

#### 4.2.2 Impact of educational attainment

Nurses holding a master’s degree or higher demonstrated significantly superior AI literacy and self-efficacy compared to other educational groups (*p* < 0.05). One possible explanation is that advanced degree programs are more likely to include AI-related content (e.g., machine learning, virtual simulation), which may contribute to a stronger knowledge base and greater operational confidence. This finding is consistent with Simms’s (28) call for the structured integration of generative AI literacy into nursing education. These results suggest that future curriculum development could consider embedding AI modules to align with the evolution of intelligent healthcare (29).

In the high-acuity PICU environment, such education may be more relevant when it is contextualized and task-driven, using clinical scenarios such as AI-assisted early warning systems or ventilator management. Additionally, fostering self-directed learning and digital literacy among nurses may help sustain their technical confidence (29, 30). These observations are consistent with Bandura’s social cognitive theory, which posits that an enriched learning environment can strengthen cognitive engagement and, in turn, cultivate positive attitudes and enhance self-efficacy. However, longitudinal studies are needed to test these potential pathways.

#### 4.2.3 Disparities between teaching and non-teaching hospitals

Nurses in teaching hospitals demonstrated higher scores in both AI attitude and self-efficacy compared to those in non-teaching hospitals (*p* < 0.05), a finding consistent with Zeng et al. (18). This pattern is consistent with Bandura’s social cognitive theory, which suggests that a resource-rich and supportive environment may play a role in cultivating positive professional behaviors and robust self-efficacy. Teaching hospitals, with their superior infrastructure and research culture, may provide more exposure to AI technologies and structured learning opportunities (33, 34).

These results suggest that addressing this disparity could involve resource sharing, collaborative training programs, and expert exchanges between teaching and non-teaching hospitals. However, the potential effectiveness of such supportive networks in facilitating knowledge transfer and system-wide capacity building requires further investigation in future longitudinal or experimental studies.

### 4.3 Interrelationships among AI attitude, AI literacy, and AI self-efficacy

The analysis revealed positive intercorrelations among the core constructs: AI attitude with AI literacy (*r* = 0.589, *p* < 0.001), AI attitude with self-efficacy (*r* = 0.533, *p* < 0.001), and most notably, AI literacy with self-efficacy (*r* = 0.743, *p* < 0.001). Willingness for AI training and use was also significantly correlated with all three variables (*p* < 0.001).

These correlations are consistent with a cognitive-behavioral pathway in which positive attitudes are associated with learning motivation, which is associated with literacy, which in turn is associated with self-efficacy and behavioral intentions. The particularly strong linkage between literacy and self-efficacy suggests that knowledge and confidence are closely related. This relationship is particularly relevant in high-stakes PICU settings, where clinical demands may require a high degree of alignment between a nurse’s knowledge, belief in their capability, and willingness to act. This pattern is consistent with the triadic reciprocal determinism posited by Bandura’s social cognitive theory (15). Our findings align with and extend existing literature. Boztepe et al. (26) established the attitude-literacy link, while Pare et al. (35) found that understanding promotes adoption. This study integrates these elements into a broader motivational framework, positioning training and use intentions as potential psychological drivers within this pathway. These findings suggest that nursing leaders could consider fostering a cycle by supporting learning motivation, foundational literacy, and applied confidence to facilitate the integration of AI into clinical practice.

### 4.4 The mediating role of AI literacy between attitude and self-efficacy

The mediation analysis showed a significant direct effect of AI attitude on self-efficacy (*β* = 0.12 in the adjusted model, accounting for approximately 24% of the total effect), suggesting that positive attitudes are associated with nurses’ confidence in using AI, a phenomenon consistent with prior research (36). Crucially, AI literacy played a notable mediating role, with the indirect pathway explaining approximately 76% of the total effect (*β* = 0.39 in the adjusted model). This suggests that the association between attitude and self-efficacy operates primarily through AI knowledge and skills. From the perspective of social cognitive theory, AI literacy could be viewed as a cognitive-affective factor within the attitude-self-efficacy sequence. Positive attitudes may be associated with a motivational cascade that supports the acquisition of AI literacy. This acquired literacy, in turn, may serve as a foundation for mastery experiences—a primary source of self-efficacy according to Bandura—which may bolster confidence and promote subsequent behavioral engagement (19).

Consistent with this view, the systematic review by Gonzalez-Garcia et al. (37) suggests that successful AI integration may depend on not merely individual readiness but also on orchestrated organizational support, underscoring the interplay between leadership initiatives and educational frameworks. Within such a supportive environment, nursing leaders could catalyze the transformation of AI literacy into self-efficacy by designing targeted training and fostering interdisciplinary collaboration.

These findings suggest that a dual-phase strategy could be considered: the integration of AI literacy into pre-service nursing curricula, complemented by ongoing, context-rich training for practicing nurses that emphasizes hands-on application and critical reflection. This could be scaffolded by a supportive learning culture and recognition systems, but these approaches require empirical testing. In resource-constrained settings like Sichuan, leveraging digital collaborative platforms and developing a repository of PICU-specific virtual AI simulation cases are potential approaches that warrant further investigation.

## 5 Implications and future directions

Given the cross-sectional design, these findings are hypothesis-generating rather than conclusive. For future research, longitudinal and intervention studies are needed to test the causal pathways suggested by the mediation model. Specifically, future research could explore whether AI literacy training programs actually lead to improvements in AI self-efficacy over time. For practice, the results suggest that nursing administrators could consider supporting AI literacy development as one potential strategy to enhance nurses’ AI self-efficacy. Previous research has indicated that nurses’ trust and adoption of AI systems may increase when AI tools are perceived as dependable, safe, and governable (38–40). However, the specific effectiveness of these approaches in the PICU context requires further investigation.

Pending confirmation from longitudinal studies, these hypothesis-generating results may inform future educational and management strategies. Nursing administrators may consider integrating AI literacy into continuing education as one potential approach, but the effectiveness of such initiatives requires empirical testing.

## 6 Limitations and future research

This study is subject to several limitations. First, the generalizability of the findings is constrained by the convenience sampling method and the geographically delimited sample, drawn exclusively from PICU nurses in public hospitals within Sichuan Province. Future studies should incorporate more diverse samples from various regions and clinical specialties to enhance external validity. Second, the cross-sectional nature of the design precludes causal inference regarding the relationships among the variables. Longitudinal or intervention-based designs are needed in subsequent research to test the proposed model’s associations. Third, all data were self-reported and collected at a single time point, which may introduce common method bias and social desirability bias, although Harman’s single-factor test indicated that common method bias was not a severe concern (first factor = 49.23%). Fourth, the subgroup with master’s degrees or above consisted of only 13 participants, and its mean estimates may be unstable. Due to this limited sample size, measurement invariance across different demographic groups (e.g., education level, gender, hospital type) was not tested. Future studies with larger and more balanced samples should examine whether the measurement properties of the scales are invariant across different groups. Fifth, although the mediation model was adjusted for education level, professional title, and teaching hospital status, other variables (e.g., AI training history) were not included as covariates. Future studies may consider adjusting for additional variables. Building on these findings, future research could develop and test a structured training intervention grounded in the attitude-literacy-self-efficacy framework. The efficacy of such an intervention in enhancing nurses’ AI readiness could be evaluated through randomized controlled trials (RCTs) or longitudinal designs. However, the present study did not test any intervention, and such evaluations remain an important direction for future research.

## Data Availability

The original contributions presented in the study are included in the article/supplementary material, further inquiries can be directed to the corresponding author.

## Footnotes

1
www.wjx.cn
